# Complete polarization control in multimode fibers with polarization and mode coupling

**DOI:** 10.1038/s41377-018-0047-4

**Published:** 2018-08-08

**Authors:** Wen Xiong, Chia Wei Hsu, Yaron Bromberg, Jose Enrique Antonio-Lopez, Rodrigo Amezcua Correa, Hui Cao

**Affiliations:** 1 Department of Applied Physics, Yale University, New Haven, CT 06520 USA; 2 Racah Institute of Physics, Hebrew University of Jerusalem, Jerusalem, 91904 Israel; 3 CREOL, The College of Optics and Photonics, University of Central Florida, Orlando, FL 32816 USA

## Abstract

Multimode optical fibers have seen increasing applications in communication, imaging, high-power lasers, and amplifiers. However, inherent imperfections and environmental perturbations cause random polarization and mode mixing, causing the output polarization states to be different from the input polarization states. This difference poses a serious issue for employing polarization-sensitive techniques to control light–matter interactions or nonlinear optical processes at the distal end of a fiber probe. Here, we demonstrate complete control of polarization states for all output channels by only manipulating the spatial wavefront of a laser beam into the fiber. Arbitrary polarization states for individual output channels are generated by wavefront shaping without constraining the input polarization. The strong coupling between the spatial and polarization degrees of freedom in a multimode fiber enables full polarization control with the spatial degrees of freedom alone; thus, wavefront shaping can transform a multimode fiber into a highly efficient reconfigurable matrix of waveplates for imaging and communication applications.

## Optics: Controlling polarization key to better performing optical fibers

By controlling the spatial wavefront of light beams, scientists have developed an innovative approach for eliminating polarization distortions in signals transmitted through optical fibers, leading to more efficiency devices for use in communication and imaging technologies. Owing to its high capacity and reliability, multimode fibers (MMFs) have seen increasing use in a range of devices used in communication, imaging, high-power lasers, and amplifiers. However, imperfections and perturbations that occur during signal transmission cause polarization scrambling and random mode mixing of the light, making the output polarization states very different from the input. Led by Hui Cao and colleagues from Yale University in the United States, researchers have developed a method for controlling polarization by utilizing strong mode and polarization coupling in the multimode fibers, which could be used for applications in optical imaging, communications and remote sensing.

## Introduction

The vectorial nature of electromagnetic waves plays an indispensable role in light–matter interaction, optical transmission, and imaging. Control over the polarization state of light has been widely exploited in single-molecule detection, nanoplasmonics, optical tweezers, nonlinear microscopy, and optical coherence tomography. However, a well-prepared state of polarization can be easily scrambled by multiple scatterings of light in three-dimensional disordered media. An alternative consideration is that multiple scatterings couple spatial and polarization degrees of freedom, enabling polarization control of the scattered light using wavefront shaping of the incident beam. Arbitrary polarization states have been attained in a single or a few spatial channels^1–5^, transforming the random medium into a dynamic waveplate. For imaging and sensing applications, full polarization control of all output channels can avoid spatial point scanning and acquire information in parallel. However, it is extremely difficult to control the polarization state of the *total* transmitted light, and the relatively low transmission through a scattering medium limits the efficiency.

**Fig. 1:**
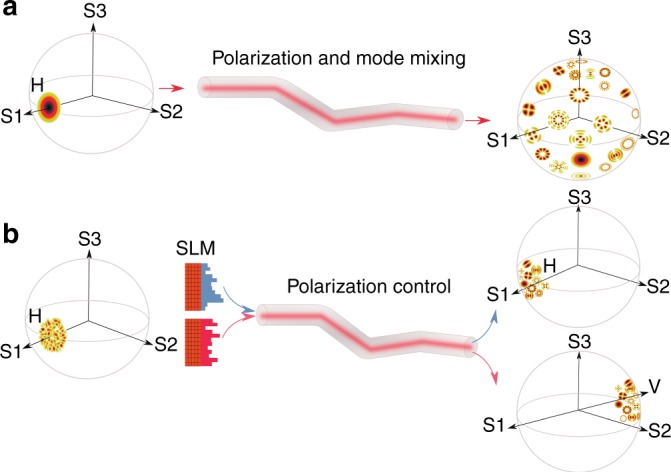
Fiber depolarization and polarization control by wavefront shaping. The three axes are the three Stokes parameters. **a** Light is launched into the fundamental LP mode with horizontal polarization and subsequently coupled to other modes with different spatial profiles and polarization states while propagating in the fiber. The transmitted light is composed of all spatial modes in different polarization states, which are randomly spread over the Poincaré sphere. **b** Wavefront shaping of the horizontally polarized light by an SLM can overcome depolarization in the fiber, retaining the horizontal polarization for all output modes (top). A different input wavefront can convert all output modes to vertical polarization (bottom)

Polarization scrambling also occurs in optical fibers^6^. For a single-mode fiber, the output polarization state can be controlled by manipulating the input polarization. Due to refractive index fluctuations introduced by inherent imperfection and environmental perturbation such as eccentricity, bending, and twisting, a multimode fiber (MMF) experiences not only polarization mixing but also mode mixing. When light is launched into a single guided mode in the MMF, it spreads to other modes, each of which experiences distinct polarization scrambling. Thus, the output polarization state of the modes varies from one mode to another (see Fig. 1a), prohibiting the control of output polarization states in *all* modes by adjusting the input polarization of a single mode. One approach to achieve complete polarization control is to measure the full transmission matrix of the MMF and invert it to find the vector fields to be injected into the individual modes. This approach requires simultaneous control of both the spatial and polarization degrees of freedom at the input and is technically demanding.

The coupling between the spatial and polarization degrees of freedom in an MMF, such as in a random scattering medium^7,8^, opens the possibility of utilizing only the spatial degrees of freedom of the input wave to control the polarization state of the output field. The key question is whether such control would be complete in the sense that arbitrary polarization states can be attained for total transmitted light regardless of the input polarization, and each output mode has a polarization state that differs from each other in a designed manner. If complete polarization control can be achieved by only shaping the spatial wavefront of an input beam, it is easier to experimentally realize because most spatial light modulators (SLMs) operate for one polarization. Complete control of output polarization states is essential in the application of MMFs in endoscopy^9–17^, spectroscopy^18–20^, microscopy^21,22^, nonlinear optics^23,24^, quantum optics^25,26^, optical communication^27^, and fiber amplifiers^28–33^.

In this study, we experimentally demonstrate complete polarization control of coherent light transmitted through an MMF with strong mode and polarization coupling. By modulating the spatial wavefront of a linearly polarized (LP) beam, depolarizations in the MMF are completely eliminated, and the transmitted light retains the input polarization. Moreover, a complete conversion of the input polarization to its orthogonal counterpart or any polarization state is achieved. We further tailor the polarization states of individual output channels utilizing spatial degrees of freedom without constraining the input polarization state. Our theoretical analysis and numerical modeling illustrate that full control of polarizations using spatial wavefront shaping is only possible when mode coupling occurs in the fiber. Random mode mixing, often unavoidable in an MMF, can be harnessed for functional advantages. Hence, wavefront shaping can make an MMF function as a highly efficient reconfigurable matrix of waveplates, converting arbitrary polarization states of the incident field into any desired polarization state.

## Results

### Mode coupling

To illustrate the critical role played by spatial mode coupling in polarization control, let us first consider an MMF with only polarization mixing but no mode mixing. Linearly polarized (LP) modes are the eigenmodes of a perfect fiber under the weak guiding approximation^34^. The birefringence induced by fiber imperfections and perturbations changes the polarization state. Light injected into each LP mode effectively propagates through a distinctive set of waveplates with random orientations of their optical axes. Eventually, different LP modes have different polarizations, and the total output field becomes depolarized. In the absence of mode coupling, the MMF behaves similar to a bundle of uncoupled single-mode fibers. It is impossible to control the output polarization of each mode without manipulating their individual input polarizations.

With mode mixing in the fiber, spatial and polarization degrees of freedom become coupled. The output polarization state depends not only on the polarization but also on the spatial wavefront of the input field. For illustration, we consider a fiber with only two modes, each of which has two orthogonal polarization states. The incident light is monochromatic and LP in the horizontal direction. The field is 1 for mode 1 and *e*^*iθ*^ for mode 2. Without mode coupling, the relative phase *θ* between the two modes does not affect the output polarization state of either mode. However, with mode coupling, the output field of one mode also depends on the input field of the other. For example, the vertical polarization of mode 1 has contributions from (i) the field in mode 1 converted to the vertical polarization and (ii) the field in mode 2 that is coupled to the vertically polarized mode 1. The relative phase of these two contributions can be changed by varying *θ*, resulting in a constructive or destructive interference that modifies the amplitude of the vertically polarized field in mode 1. This degree of freedom is effective only when there is mode mixing in the fiber. Compared to a fiber without mode coupling, more polarization states can be created at the output by adjusting the input wavefront. Mode mixing enables polarization control by utilizing spatial degrees of freedom, as illustrated schematically in Fig. 1b.

### Polarization manipulation

#### Depolarization-free states

To quantitatively evaluate the polarization control using only spatial degrees of freedom, we perform a numerical simulation of an MMF with strong polarization and mode coupling. The fiber has *N* modes, each of which has a two-fold degeneracy corresponding to two orthogonal polarizations. We use the concatenated fiber model^35^ to simulate random coupling among all modes of the MMF (see Supplementary Materials). Without loss of generality, we use the horizontal (H) and vertical (V) polarizations as the basis to describe the full transmission matrix of the MMF

1 $$t = \left[ {\begin{array}{*{20}{c}} {t_{{\mathrm{HH}}}} & {t_{{\mathrm{HV}}}} \\ {t_{{\mathrm{VH}}}} & {t_{{\mathrm{VV}}}} \end{array}} \right]$$

where *t*_HH_(*t*_VH_) represents the horizontal (vertical) component of the output field when the input light is horizontally polarized. *t*_HH_ has a dimension of *N* × *N*, where *N* is the number of modes in the fiber for a single polarization.

**Fig. 2:**
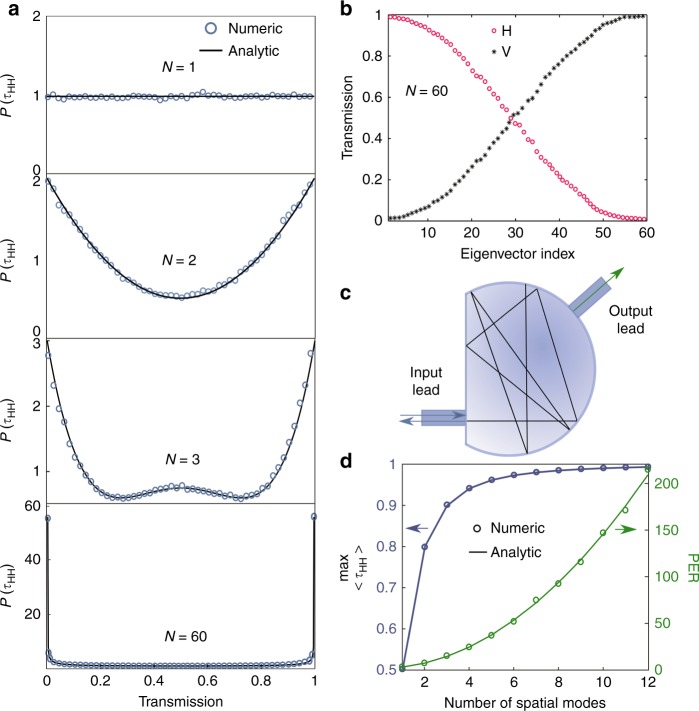
Polarization mixing in an MMF and analogy to wave scattering in a chaotic cavity. **a** Numerically calculated density of the eigenvalues of $t_{{\mathrm{HH}}}^\dagger t_{{\mathrm{HH}}}$ (blue circles) in an MMF with strong mode and polarization coupling. With an increasing number of modes *N* in the fiber, *P*(*t*_HH_) evolves into a bimodal distribution, in agreement with the analytical expression for the reflection eigenvalue density in a chaotic cavity with two leads (black so $\tau _{{\mathrm{HH}}}^{{\mathrm{max}}}$ lid lines). **b** Transmission of horizontal (H) and vertical (V) polarization components for individual eigenvectors of $t_{{\mathrm{HH}}}^\dagger t_{{\mathrm{HH}}}$, which are numbered by their eigenvalues from high to low. The decrease of H is accompanied by an increase of V, and their sum remains 1. **c** Schematic diagram of a chaotic cavity with two leads. A wave enters the cavity through the input lead and undergoes multiple reflections from the cavity wall before exiting through the output lead (transmission) or the input lead (reflection). **d** The maximum transmission of horizontal polarization approaches 1 rapidly with increasing *N*. The *PER* scales as *N*^2^. The symbols represent numerical data and the solid lines are the analytical results

The output field of the horizontal polarization is $\left| \psi \right\rangle = t_{{\mathrm{HH}}}\left| \phi \right\rangle$ for a horizontally polarized input $\left| \phi \right\rangle$. Hence, the total intensity of the horizontal polarization is $\left\langle {\psi |\psi } \right\rangle = \left\langle {\phi \left| {t_{{\mathrm{HH}}}^\dagger t_{{\mathrm{HH}}}} \right|\phi } \right\rangle$, with $t_{{\mathrm{HH}}}^\dagger$ being the Hermitian conjugate of *t*_HH_. The maximum and minimum eigenvalues of $t_{{\mathrm{HH}}}^\dagger t_{{\mathrm{HH}}}$ set the range of transmission that can be reached in horizontal polarization. The largest eigenvalue gives the maximum energy that can be retained in the horizontal polarization after propagating through the fiber. In contrast, the smallest eigenvalue indicates the maximum energy that can be converted to the vertical polarization. After simulating an ensemble of MMFs with random mode and polarization coupling but no loss, we obtain the eigenvalue density *P*(*τ*_HH_) plotted in Fig. 2a. If the fiber has only one mode (*N* = 1), *P*(*τ*_HH_) has a uniform distribution between 0 and 1 as a result of complete polarization mixing in the fiber. When there are two guided modes (*N* = 1), *P*(*τ*_HH_) develops two peaks at *τ*_HH _= 0, 1. With the increase in *N*, these two peaks grow rapidly and become dominant at $N \gg 1$. The *P*(*τ*_HH_) probability of having an eigenvalue close to unity or zero is very high (see Supplementary Materials). The eigenvector associated with *τ*_HH_ = 1 retains the input polarization (H) at the fiber output, whereas the eigenvector associated with *τ*_HH_ = 0 makes a 100% conversion to the orthogonal polarization (V). As *τ*_HH_ decreases from 1 to 0, the percentage of transmission in the horizontal polarization drops, whereas that in the vertical polarization rises, as shown in Fig. 2b.

The numerically calculated eigenvalue density agrees with the analytical prediction of wave transmission in a lossless chaotic cavity (see the lines in Fig. 2a). Such an agreement reveals the analogy between an MMF with random mode and polarization coupling and a chaotic cavity with two leads, as drawn schematically in Fig. 2c. The transmission of the input polarization in the fiber is analogous to the reflection in the chaotic cavity in the sense that light exits the cavity through the same lead. Hence, the eigenvalue for the MMF corresponds to the reflection eigenvalue of the chaotic cavity.

Using the analytical theory developed previously for the chaotic cavity^36,37^, we derive the probability density of the maximum *τ*_HH_ eigenvalue of $t_{{\mathrm{HH}}}^\dagger t_{{\mathrm{HH}}}$ (see Supplementary Materials). The average value, $\left\langle {\tau _{{\mathrm{max}}}} \right\rangle = 1 - 1{\mathrm{/}}(N^2 + 1)$, approaches unity rapidly with the increase in *N*, in agreement with the numerical result shown in Fig. 2d. The polarization extinction ratio (PER), defined as the maximal ratio of the transmissions in the two orthogonal polarizations, is equal to$\left\langle {\tau _{{\mathrm{max}}}} \right\rangle {\mathrm{/}}(1 - \left\langle {\tau _{{\mathrm{max}}}} \right\rangle ) = N^2$. With a large number of modes in the fiber, we obtain . Depolarization is avoided by coupling light with the eigenvector associated with the maximum eigenvalue of $t_{{\mathrm{HH}}}^\dagger t_{{\mathrm{HH}}}$. The eigenvector is a superposition of the LP modes with the horizontal polarization and can be generated by an SLM. The *N*^2^ scaling originates from the repulsion between eigenvalues, leading to the bimodal distribution of eigenvalues^8,38^.

For comparison, we consider the scaling of PER in an MMF without mode mixing. Due to different polarization coupling for individual modes, the probability of retaining the input polarization for all output modes vanishes when the number of modes is large. The best solution to retain the input polarization is to excite the mode with an output polarization closest to the input polarization. As detailed in Supplementary Materials, the PER of the transmitted light scales linearly with *N*, inferior to the *N*^2^ scaling in the presence of strong mode coupling. This comparison shows that spatial mode mixing greatly enhances the ability of overcoming depolarization.

The above results are obtained when the fiber has negligible loss. If the loss in the fiber is significant, the eigenvalue density is modified, and the maximum eigenvalue is <1. Consequently, the PER for the eigenvector associated with the maximum eigenvalue of $t_{{\mathrm{HH}}}^\dagger t_{{\mathrm{HH}}}$ is reduced. As shown in the Supplementary Materials, regardless of how strong the loss is, the PER of an MMF with mode coupling is always higher than that without mode coupling. Therefore, mode coupling enhances the polarization control regardless of the loss in the fiber. Furthermore, complete polarization control can still be achieved even when the fiber undergoes significant loss, as described in the next subsection.

#### Polarization conversion

The efficiency of converting the input polarization (H) to the orthogonal polarization (V) is given by the minimum eigenvalue of $t_{{\mathrm{HH}}}^\dagger t_{{\mathrm{HH}}}$. When loss in the MMF is negligible, the minimum eigenvalue of $t_{{\mathrm{HH}}}^\dagger t_{{\mathrm{HH}}}$ and the maximum eigenvalue of $t_{{\mathrm{VH}}}^\dagger t_{{\mathrm{VH}}}$ correspond to the same eigenvector because $t_{{\mathrm{HH}}}^\dagger t_{{\mathrm{HH}}} + t_{{\mathrm{VH}}}^\dagger t_{{\mathrm{VH}}} = 1$. When the polarizations are completely mixed, the transmitted field has no memory of its initial polarization; thus, the transmission matrix *t*_VH_ has the same statistical property as *t*_VH_. The eigenvalue density *P*(*τ*_VH_) is identical to *P*(*τ*_HH_) and has a bimodal distribution. We obtain the ensemble-averaged value $\left\langle {\tau _{{\mathrm{HH}}}^{{\mathrm{min}}}} \right\rangle = 1 - \left\langle {\tau _{{\mathrm{VH}}}^{{\mathrm{max}}}} \right\rangle = 1/(N^2 + 1)$ and PER = *N*^2^. In Supplementary Materials, we provide the analytical expression for its probability density $P(\tau _{{\mathrm{HH}}}^{{\mathrm{min}}})$. When $N \gg 1$, light is almost completely transformed into the orthogonal polarization by spatially coupling light to the eigenvector of $t_{{\mathrm{HH}}}^\dagger t_{{\mathrm{HH}}}$ with the minimum eigenvalue.

If the fiber suffers significant loss, the maximum eigenvalue becomes <1, but the minimum eigenvalues remain close to 0. The eigenvector associated with the minimum eigenvalues can be used for complete polarization control, despite the reduced total transmission. For example, if the input light is horizontally polarized, by coupling it to the eigenvector of $t_{{\mathrm{VH}}}^\dagger t_{{\mathrm{VH}}}$ with an eigenvalue close to 0, the transmitted light has a vanishing vertical component. Thus, depolarization is avoided, but part of the incident light is lost instead of being transmitted. Additionally, the transmitted light can be converted to vertical polarization by exciting the eigenvector of $t_{{\mathrm{HH}}}^\dagger t_{{\mathrm{HH}}}$ with the minimum eigenvalue.

Thus far, we have considered only the horizontal and vertical polarizations for the input and output fields, but the same concept applies to any polarization state. As long as the polarization of light is completely scrambled in the fiber, all polarization states are equivalent. For example, let us consider the conversion from the horizontally polarized (H) input to the right-hand circular polarized (R) output. The corresponding transmission matrix $t_{{\mathrm{RH}}}^\dagger t_{{\mathrm{RH}}}$ has the same eigenvalue density as $t_{{\mathrm{HH}}}^\dagger t_{{\mathrm{HH}}}$. With strong mode coupling and negligible loss in the fiber, *P*(*τ*_RH_) is bimodal, and the peak at *τ*_RH_ = 1(*τ*_RH_ = 0) allows full conversion of the horizontal polarization to right (left) circular polarization.

#### Multi-channel polarization transformation

**Fig. 3:**
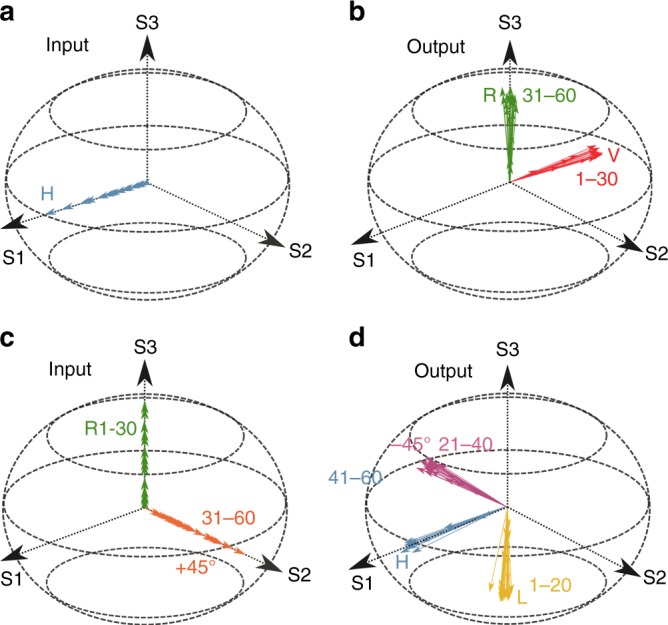
Poincaré sphere representation of multi-channel polarization transformation in the MMF. The direction of each arrow stands for the polarization of each mode and the length represents the intensity of the mode. **a**, **b** Transformation of the input horizontal polarization (H) into the output polarization state with vertical polarization (V) for modes 1–30 and right-hand circular polarization (R) for modes 31–60. **c**, **d** Transformation of the input polarization state with right-hand circular polarization (R) for modes 1–30 and linear +45° polarization for modes 31–60 into the output polarization state with left-hand circular polarization (L) for modes 1–20, linear −45° polarization for modes 21–40, and horizontal polarization (H) for modes 41–60

Let us take one step further: instead of controlling the polarization state of the total transmission, we can have different polarization states for different modes. As an example, we transform the horizontal polarization (H) of the input field (Fig. 3a) into a complex polarization state (A) at the output of an MMF with 60 modes. As shown in Fig. 3b, the polarization state A has the vertical polarization (V) for modes 1–30 and right-hand circular polarization (R) for modes 31–60. The conversion is achieved by coupling the incident light to the eigenvector of $t_{{\mathrm{AH}}}^\dagger t_{{\mathrm{AH}}}$ with the eigenvalue close to 1, when the loss in the fiber is negligible. When the loss is significant, we resort to the output polarization state B that is orthogonal to A. In this case, B has horizontal polarization (H) for modes 1–30 and left-hand circular polarization (L) for modes 31–60. By exciting the eigenvector of $t_{{\mathrm{BH}}}^\dagger t_{{\mathrm{BH}}}$ with the eigenvalue close to 0, the output polarization state is orthogonal to B and thus identical to A.

Finally, our scheme can also handle arbitrary input polarization states; that is, individual spatial modes can have different polarizations at the fiber input. By adjusting the input spatial wavefront, arbitrary polarizations can be obtained at the output. See the Supplementary Materials for a detailed illustration of multi-channel polarization transformation. Figure 3c, d presents one example. Therefore, an MMF with strong mode and polarization coupling is capable of transforming arbitrary input polarizations into arbitrary output polarizations with nearly 100% efficiency. Because only the spatial degrees of freedom are deployed at the input, the output intensity in each spatial mode, that is, the distribution of output energy among spatial modes, cannot be controlled. To design not only the polarizations but also the intensities of all output modes, both the spatial and polarization degrees of freedom at the input are utilized.

### Experimental demonstration

**Fig. 4:**
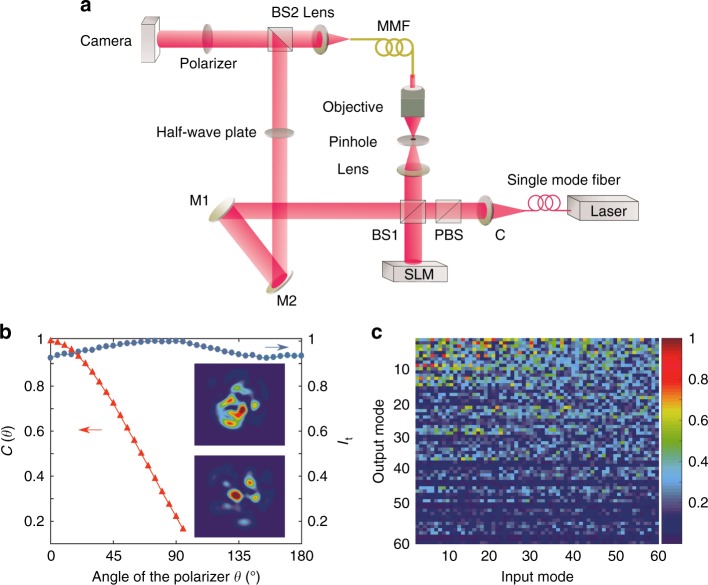
Experimental setup and fiber calibration. **a** Schematic of the interferometric setup for measuring the transmission matrix of an MMF. **b** Characterization of depolarization in the MMF. The total transmitted intensity *I*_t_ (blue dots) and the correlation of output intensity patterns *C*(*θ*) (red triangles) confirm complete depolarization. *θ* is the angle of the polarizer. Insets: intensity patterns of orthogonal polarizations measured when *θ* = 0° and 90°. **c** Amplitude of measured transmission matrix *t*_HH_ in the LP mode basis reveals strong mode mixing in the fiber. C collimator, BS beam splitter, PBS polarizing beam splitter, M mirror

We experimentally demonstrate complete polarization control of an MMF with strong polarization and mode coupling by wavefront shaping. We characterize the polarization-resolved transmission matrix with an interferometric setup shown in Fig. 4a. A detailed description can be found in the Materials and methods section. To quantify the depolarization in the MMF, we measure the total transmitted intensity *I*_t_ as a function of the angle of linear polarizer *θ*. As shown in Fig. 4b, *I*_t_ only exhibits slight (~9%) variations with *θ*. Furthermore, the output intensity pattern changes with *θ*, and thus, the individual output channels have distinct polarizations. We compute the correlation function $C(\theta ) = \vec I(0) \cdot \vec I(\theta )$, where $\vec I(\theta )$ is a unit vector representing the normalized intensity profile at *θ*. The decay of *C*(*θ*) in Fig. 4b illustrates the decreasing correlation of the intensity pattern with *θ*. The insets of Fig. 4b are the two intensity patterns of the orthogonal polarizations (*θ* = 0, 90°), which are almost uncorrelated, indicating nearly complete depolarization.

The amplitude of the measured transmission matrix *t*_HH_ in the LP mode basis is shown in Fig. 4c. Regardless of which mode light is injected into, the output field spreads over all modes, although higher order modes have lower amplitudes due to stronger dissipations. The measured *t*_VH_, given in the Supplementary Materials, has similar characteristics. These results confirm strong spatial and polarization mixing in the MMF.

**Fig. 5:**
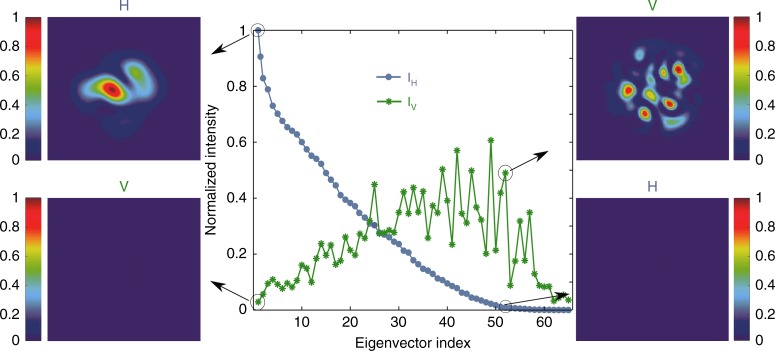
Experimental demonstrations of overcoming fiber depolarization and complete conversion to orthogonal polarization. Central panel: intensities of the horizontal (H) and vertical (V) polarization components of total transmitted light *I*_H_ and *I*_V_ for individual eigenvectors of the experimentally measured $t_{{\mathrm{HH}}}^\dagger t_{{\mathrm{HH}}}$. The eigenvectors are arranged by *I*_H_ from high to low, and the largest value of *I*_H_ is normalized to 1. The experimentally measured output intensity patterns of H and V for the 1st and the 52nd eigenvectors are shown on the left and right, respectively

To control the output polarization, we compute the eigenvectors of the experimentally measured $t_{{\mathrm{HH}}}^\dagger t_{{\mathrm{HH}}}$. For each eigenvector, the intensities of the horizontal and vertical polarization components in the total transmitted light *I*_H_ and *I*_V_ are plotted in Fig. 5. The first eigenvector is associated with the largest eigenvalue, thus having the maximum *I*_H_ and the minimum *I*_V_. The eigenvectors are ordered by the value of *I*_H_ from high to low. The decrease in *I*_H_ is accompanied by the increase in *I*_V_. Eventually, *I*_V_ cannot reach the maximum of *I*_H_ due to mode-dependent loss in the fiber. Employing the computer-generated phase hologram for a simultaneous phase and amplitude modulation^39^, we create the input wavefront for the first eigenvector with the SLM and launch it into the fiber. The output intensity patterns of the horizontal and vertical polarizations are recorded (left panel in Fig. 5). Because higher order modes suffer more loss in the fiber, the transmitted light is mainly composed of lower order modes. The horizontal polarization component is much stronger than the vertical one, and the PER is approximately 24. Hence, most of the energy is retained in the input polarization (H), and depolarization is overcome. The experimentally obtained PER is in agreement with the numerical simulation result of a fiber with a comparable amount of loss (see the Supplementary Materials).

A complete conversion to orthogonal polarization (V) is achieved with the eigenvector of $t_{{\mathrm{HH}}}^\dagger t_{{\mathrm{HH}}}$ with a small eigenevalue. For example, we choose the 52nd eigenvector, which has a low transmission of the horizontal polarization, and launch its input field profile into the MMF. The measured output intensity patterns are shown in the right panel of Fig. 5, where the transmitted light is dominated by the vertical polarization component. The PER is 43, exceeding that of the first eigenvector. Because the 52nd eigenvector has more contributions from the higher order modes, which undergo a higher attenuation than that of the lower order modes, its transmission is approximately half that of the first eigenvector.

**Fig. 6:**
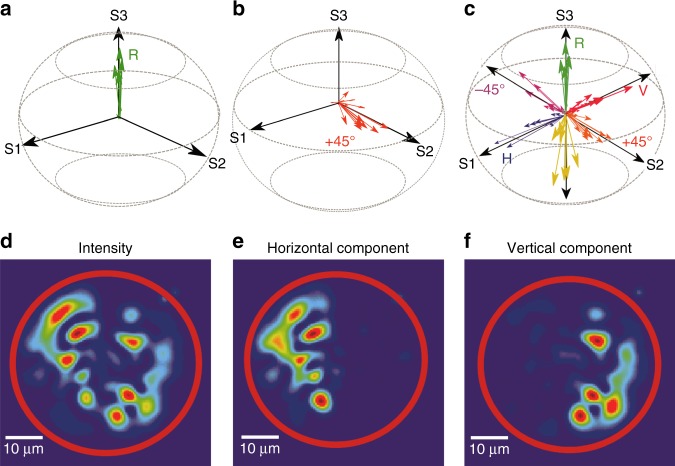
Experimental generation of arbitrary polarization states. **a**–**c** Poincaré sphere representation of output polarization state of **a** the highest transmission eigenchannel of $t_{{\mathrm{RH}}}^\dagger t_{{\mathrm{RH}}}$, all LP modes (each represented by an arrow) are right circularly polarized, **b** a low transmission eigenchannel of $t_{ - 45{\mathrm{H}}}^\dagger t_{ - 45{\mathrm{H}}}$, all modes are linearly polarized in the +45° direction. **c** different polarization states generated with a fixed input polarization (H). **d**–**f** Output intensity pattern (**d**), its horizontal (**e**) and vertical (**f**) polarization components reveal that the transmitted field in the left half of the fiber facet is horizontally polarized and the right half vertically polarized

We can convert the horizontally polarized light to any polarization state at the fiber output. For example, to obtain right circular polarization (R), we construct $t_{{\mathrm{RH}}} = \frac{1}{{\sqrt 2 }}(t_{{\mathrm{HH}}} - {\mathrm {i}}t_{{\mathrm{VH}}})$ from the measured *t*_HH_ and *t*_VH_ and couple the incident light to the eigenvector of $t_{{\mathrm{RH}}}^\dagger t_{{\mathrm{RH}}}$ with the largest eigenvalue. The output polarization states of the individual LP modes are measured and plotted on a Poincaré sphere, as shown in Fig. 6a. Each arrow represents one mode, and its length stands for the intensity of that mode. All arrows point along the *S*_3_ axis, indicating that all modes are circularly polarized, despite varying intensities. In Fig. 6b, we obtain linear +45° polarization by exciting a low transmission eigenchannel of $t_{ - 45{\mathrm{H}}}^\dagger t_{ - 45{\mathrm{H}}}$, where $t_{ - 45{\mathrm{H}}} = \frac{1}{{\sqrt 2 }}\left( {t_{{\mathrm{HH}}} - t_{{\mathrm{VH}}}} \right)$. Figure 6c shows different polarization states that are experimentally generated with a fixed input polarization (H).

To demonstrate complete polarization control, we create different polarization states for the individual output channels. In addition to the fiber mode basis, the spatial channels can be represented in real space (near-field zone of the fiber distal end) or momentum space (far-field zone). In the following example, we describe the fiber output channels in real space. The output polarization state (C) is designed to have horizontal polarization for the spatial channels within the left half of the fiber cross-section and vertical polarization in the right half. The transmission matrix *t*_CH_ is constructed by concatenating one half of *t*_HH_ and the other half of *t*_VH_. The conversion of polarization from H to C is realized by exciting the highest transmission eigenchannel of $t_{{\mathrm{CH}}}^\dagger t_{{\mathrm{CH}}}$. Figure 6d is an image of intensity pattern at the fiber output facet taken by a camera without a polarizer. After the linear polarizer is placed in front of the camera and oriented in the horizontal direction, the right half becomes dark whereas the left half remains bright in Fig. 6e. Once the polarizer rotates in the vertical direction, the right half lights up whereas the left half turns dark in Fig. 6f. Hence, the transmitted field is horizontally polarized in the left half of the fiber facet and vertically polarized in the right half. An additional example is given in Supplementary Materials, which shows that the output field is the left-hand circular polarization (L) in the top half of the fiber facet and right-hand circular polarization (R) in the bottom half.

## Discussion

Random mode mixing has been regarded as an obstacle for MMF applications, and there have been intensive efforts to reduce or eliminate mode coupling. Instead of battling it, we take advantage of mode mixing for polarization control in a fiber. We demonstrate that strong coupling between the spatial and polarization degrees of freedom in an MMF enable complete control of the output polarization states by manipulating only the spatial input wavefront. A general procedure of finding the spatial wavefront to create an arbitrary polarization state is outlined and experimentally confirmed. The procedure involves the measurement of the polarization-resolved transmission matrix and a selective excitation of the transmission eigenchannels corresponding to the extremal eigenvalues. With the random mixing of all the modes of the different polarizations in the fiber, the probability of having extremal eigenvalues is enhanced by eigenvalue repulsion, which is analogous to a chaotic cavity. We apply the existing theory of chaotic cavities to MMFs, uncovering the connection between the two fields of wave chaos and fiber optics.

The global control of polarization states for MMFs is not only useful for overcoming depolarization in an MMF but also valuable for employing polarization-sensitive imaging techniques of fiber endoscopy and nonlinear microscopy. In this work, we demonstrate polarization control for monochromatic light, which is relevant, for example, to fiber-based fluorescence microscopy with laser excitation. Our scheme of MMF polarization control is applicable at any wavelength, but the input wavefront for a specific output polarization state is wavelength-dependent. The polarization-shaping channels have a finite bandwidth, which corresponds to the spectral correlation width of the MMF (see Supplementary Materials). Nonlinear optics applications often use broad-band short pulses and require a large spectral bandwidth for polarization-shaping channels, which can be achieved with MMFs that have small differential group delay.

## Materials and methods

We experimentally test different types of MMFs and obtain similar results. The fiber whose data are presented in Figs. 4–6 has the graded refractive index profile designed to reduce mode-dependent loss (see Supplementary Materials), leading to the highest degree of polarization control. The core diameter of the fiber is 50 *μ*m, and the numerical aperture (NA) is approximately 0.22. The fiber is 2 m long. To enhance mode and polarization mixing in the MMF, the bare fiber is coiled to 5 loops (without a spool) and pressed by 12 clamps that are arranged in a circle. The clamps not only introduce mode and polarization coupling at multiple points in the fiber but also stabilize the fiber (see Supplementary Materials). We characterize the polarization-resolved transmission matrix with an interferometric setup shown in Fig. 4a. A horizontally polarized laser beam at wavelength *λ* = 1550 nm is split into a fiber arm and a reference arm. The SLM in the fiber arm prepares the spatial wavefront of light before it is launched into the MMF. To measure the field transmission matrix, plane waves with different wavevectors, covering the range of the fiber NA, are projected onto the input facet of the fiber. The output facet of the fiber is directly imaged by a lens onto a camera. A linear polarizer in front of the camera filters out the polarization component of light transmitted through the fiber. By rotating the linear polarizer, we measure different polarization components of the fiber output. A half-waveplate in the reference arm rotates the polarization direction of the reference beam to match the direction of the linear polarizer. The reference beam then combines with the fiber output beam at a beam splitter, and their interference fringes are recorded by a camera. Using off-axis holography, we extract the amplitude and phase of the field exiting the fiber with the same polarization as the reference^40^. The transmission matrix is measured with the input in the momentum (wavevector) basis and the output in real space. Then, we perform a basis transformation to represent the matrix in the fiber LP mode basis, as shown in Fig. 4c. By rotating the linear polarizer and the half-waveplate, the transmission matrices for both the horizontal and vertical polarizations are measured. After computing the eigenvalues and eigenvectors of the transmission matrix, we generate the input wavefronts of the individual eigenvectors with the SLM. To modulate both the amplitude and phase of the input field with a phase-only SLM, a computer-generated phase hologram is employed, and a pinhole on the back focal plane of the lens in the fiber arm filters out the first order diffraction pattern of the SLM^39^.

## Electronic supplementary material

Light: Science & Applications

Complete Polarization Control in Multimode Fibers with Polarization and Mode Coupling: Supplementary Information
