# Author Correction: Complete polarization control in multimode fibers with polarization and mode coupling

**DOI:** 10.1038/s41377-020-0316-x

**Published:** 2020-04-29

**Authors:** Wen Xiong, Chia Wei Hsu, Yaron Bromberg, Jose Enrique Antonio-Lopez, Rodrigo Amezcua Correa, Hui Cao

**Affiliations:** 1 Department of Applied Physics, Yale University, New Haven, CT 06520 USA; 2 Racah Institute of Physics, Hebrew University of Jerusalem, Jerusalem, 91904 Israel; 3 CREOL, The College of Optics and Photonics, University of Central Florida, Orlando, FL 32816 USA

**Keywords:** Optical physics, Optical manipulation and tweezers

Correction to: *Light: Science & Application*

10.1038/s41377-018-0047-4 published online 8 August 2018

We would like to correct an incomplete sentence in the last paragraph on page 3.

“With a large number of modes in the fiber, we obtain.” should be corrected to

“With a large number of modes in the fiber, we obtain PER >> 1.”

We would like to apologize for any confusion this may have caused.

