# Supplementary material for: Complete polarization control in multimode fibers with polarization and mode coupling
Source: Light Sci Appl. 2018 Aug 8;7:54. doi: 10.1038/s41377-018-0047-4 (PMC6106992; doi:10.1038/s41377-018-0047-4)
Supplement: Supplementary file 1 — Light: Science & Applications [file 41377_2018_47_MOESM1_ESM.docx]

***Light: Science & Applications***

# Optics: Controlling polarization key to better performing optical fibers

By controlling the spatial wavefront of light beams, scientists have developed an innovative approach for eliminating polarization distortions in signals transmitted through optical fibers, leading to more efficiency devices for use in communication and imaging technologies. Owing to its high capacity and reliability, multimode fibers (MMF) have seen increasing use in a range of devices used in communication, imaging, high-power lasers and amplifiers. However, imperfections and perturbations that occur during signal transmission cause polarization scrambling and random mode mixing of the light, making the output polarization states very different from the input. Led by Hui Cao and colleagues from Yale University in the United States, researchers have developed a method for controlling polarization by utilizing strong mode and polarization coupling in the multimode fibers, which could be used for applications in optical imaging, communications and remote sensing.

**Related article manuscript number:**  LSA20180055RR

**Article title:** Complete Polarization Control in Multimode Fibers with Polarization and Mode Coupling

**Corresponding author and affiliation/s:** Hui Cao, Department of Applied Physics, Yale University, New Haven, Connecticut 06520, USA

**About your Editorial Summary — please read**

**Before approving this Editorial Summary, please carefully check that (1) the summary text lists the correct author(s) and (2) the spelling and order of all author names and affiliations are correct.**

This **Editorial Summary** is based on your manuscript that was recently accepted for publication in *Light: Science & Applications* (LSA). It provides a non-specialist audience with a synopsis of your key research outcomes and conclusions. This value-added service provided by Springer Nature is designed to raise interest in your research across the broader community.

Springer Nature will publish the summary on the journal’s website, and it will be freely available under the CC BY licence (Creative Commons Attribution v4.0 International Licence) (see the journal website for details). We encourage you to re-use the summary to bring attention to your research; for example, you can host it on your own website and share it via social-networking platforms. Please attribute the summary to *LSA* and your article (e.g. by providing a link to your article) and do not make derivatives.

Please note that to maximise the usefulness of these summaries they must follow several stringent guidelines:
-- Spelling, punctuation and style are set according to *Nature* editorial guidelines. As this summary is aimed at non-expert readers, some concepts and technical terms will be simplified.
-- Total length must be no more than 135 words. It is likely that not all points in the paper will be covered.
-- The first sentence must be no more than 280 characters, including spaces, to allow use on microblogging sites.
-- The headline must consist of a brief generic subject identifier followed by a short description. No more than 10 words in total.

Please contact the editorial office () immediately with corrections should you find any factual errors in this Editorial Summary.
