# Supplementary material for: Complete polarization control in multimode fibers with polarization and mode coupling
Source: Light Sci Appl. 2018 Aug 8;7:54. doi: 10.1038/s41377-018-0047-4 (PMC6106992; doi:10.1038/s41377-018-0047-4)
Supplement: Supplementary file 2 — Complete Polarization Control in Multimode Fibers with Polarization and Mode Coupling: Supplementary Information [file 41377_2018_47_MOESM2_ESM.pdf]

Complete Polarization Control in Multimode Fibers with Polarization and Mode Coupling: Supplementary Information

Wen Xiong,[1] Chia Wei Hsu,[1] Yaron Bromberg,[2] Jose Enrique Antonio-Lopez,[3] Rodrigo Amezcua Correa,[3] and Hui Cao[1]

[1]*Department of Applied Physics, Yale University, New Haven, Connecticut 06520, USA*
[2]*Racah Institute of Physics, Hebrew University of Jerusalem, Jerusalem 91904, Israel*
[3]*CREOL, The College of Optics and Photonics, University of Central Florida, Orlando, Florida 32816, USA*

I. CONCATENATED FIBER MODEL

Experimentally we use 12 clamps to apply stress at multiple points on the fiber. This can be simulated by the concatenated fiber model [1]. The fiber is two-meter long and divided into 5 segments. The numerical aperture (NA) is 0.22. By varying the core diameter, we change the number of spatial modes. Each spatial mode has a two-fold degeneracy corresponding to horizontal (H) and vertical (V) linear polarizations. Light propagates without polarization or mode coupling in each segment. Between adjacent segments, all modes of different spatial profiles and polarizations are randomly coupled, which is modeled by a unitary random matrix. The total transmission matrix is the product of the transmission matrix for each segment.

As long as there is strong mode and polarization coupling in each segment, the exact number of segments in the concatenated model does not affect the degree of polarization control. To confirm this, we calculate the PER of the fiber output using the concatenated model with different number of fiber segments. As shown in Fig. S1, the PER does not change with the number of segments, because one segment already introduces complete polarization and mode mixing.

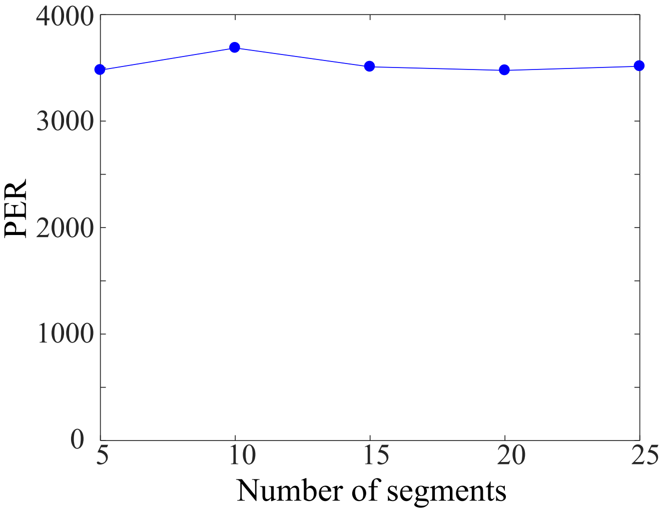

FIG. S1: Numerical simulation of PER at the output of an MMF with different number of segments. The MMF has 60 spatial modes, the PER is independent of the number of fiber segments, as long as there is complete polarization and mode mixing in a single segment.

II. FIBER TRANSMISSION MATRIX

If loss in a multimode fiber (MMF) is negligible, the full transmission matrix for both polarizations

$$t = \begin{bmatrix} t_{\mathrm{HH}} & t_{\mathrm{HV}} \\ t_{\mathrm{VH}} & t_{\mathrm{VV}} \end{bmatrix}$$

is a random unitary matrix of dimension $2N \times 2N$, where N is the number of spatial modes for a single polarization in the fiber. With strong polarization and mode coupling, such a matrix t has no symmetry other than unitarity and is a member of the circular unitary ensemble (CUE) [2]. Since t_{HH} and t_{VH} are two statistically equivalent quarters of the full matrix t, they have identical statistical properties. Their eigenvalue density evolves to a bimodal distribution at large N.

III. CHAOTIC CAVITY

The transmission matrix t for a lossless MMF with strong polarization and mode coupling is mathematically analogous to the scattering matrix s of a lossless chaotic cavity with two leads [see Fig.2 (c) of the main text]. Wave enters the chaotic cavity through one lead, then reflected multiple times from the cavity wall before escaping via the same lead or the other lead. Each lead is a waveguide with N statistically equivalent channels. The four components of the scattering matrix

$$s = \begin{bmatrix} r_1 & t_2 \\ t_1 & r_2 \end{bmatrix}$$

correspond to transmissions and reflections at the two leads. When reciprocity is broken (e.g., via magnetic field), $t_1 \neq t_2^{\mathrm{T}}$, and the s matrix is a member of CUE. Both $r_{1,2}$ and $t_{1,2}$ are statistically equivalent $N \times N$ matrices. The density of transmission or reflection eigenvalues exhibits a bimodal distribution, $p(\tau) = 1/\pi\sqrt{\tau(1-\tau)}$, for large N [3].

IV. MAXIMUM TRANSMISSION EIGENVALUE

The joint probability density for the N eigenvalues of $t_{\mathrm{HH}}^{\dagger}t_{\mathrm{HH}}$ or $t_{\mathrm{VH}}^{\dagger}t_{\mathrm{VH}}$ for the MMF, $\{\tau_1,\ldots,\tau_N\}$, is identical to the joint probability of reflection or transmission eigenvalues of a chaotic cavity, which is [3, 4]

$$p(\tau_1,\ldots,\tau_N)=c_N\prod_{n<m}(\tau_n-\tau_m)^2, \qquad (1)$$

with $\tau_n\in(0,1)$ for all n. Here c_N is a normalization constant such that $\int(\prod_{n=1}^{N}d\tau_n)p(\tau_1,\ldots,\tau_N)=1$ and $\prod_{n<m}$ is short for $\prod_{n=1}^{N}\prod_{m=n+1}^{N}$. The reduced probability when two eigenvalues are close by is a result of eigenvalue repulsion [2].

Let $\tau_{\max}$ be the largest among the N eigenvalues. The probability density of $\tau_{\max}$ follows from (1) as

$$p(\tau_{\max})=N\int_0^{\tau_{\max}}d\tau_1\cdots\int_0^{\tau_{\max}}d\tau_{N-1}p(\tau_1,\ldots,\tau_{N-1},\tau_{\max}). \qquad (2)$$

The integrals in (2) gives

$$p(\tau_{\max})=N^2(\tau_{\max})^{N^2-1},\quad \tau_{\max}\in(0,1). \qquad (3)$$

Similarly, the probability density for the smallest eigenvalue is $p(\tau_{\min})=N^2(1-\tau_{\min})^{N^2-1}$.

Figure S2 plots the probability of having at least one eigenvalue above 0.95. The probability increases dramatically with N and reaches 1 for $N\geqslant 10$.

From Eq.3, we get

$$\langle\tau_{\max}\rangle=1-\frac{1}{N^2+1},$$
$$\mathrm{var}\,(\tau_{\max})=\frac{N^2}{(N^2+1)^2(N^2+2)}. \qquad (4)$$

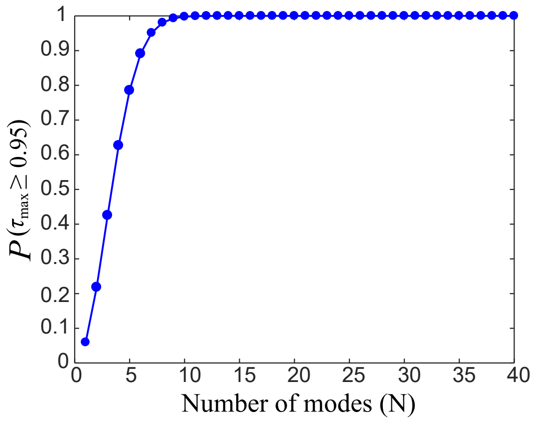

FIG. S2: Probability of finding at least one eigenvector of $t_{\mathrm{HH}}^{\dagger}t_{\mathrm{HH}}$ with an eigenvalue (transmission in horizontal polarization) exceeding 0.95.

In the absence of loss, both $1-\langle\tau_{\max}\rangle$ and the standard deviation of $\tau_{\max}$ scale as $1/N^2$ for large N. This is because the eigenvalues near 1 are pushed further toward 1 by the repulsion from the smaller eigenvalues and there are no eigenvalues larger than 1 to counter balance this push. We define the polarization extinction ratio (PER) as the maximal ratio of the transmissions in the two orthogonal polarizations, $\langle\tau_{\max}\rangle/(1-\langle\tau_{\max}\rangle)=N^2$.

V. POLARIZATION CONTROL WITHOUT MODE MIXING

With negligible mixing between different spatial modes in the fiber, each pair of LP modes with same spatial profile and orthogonal polarization is coupled due to birefringence. Light injected to a spatial mode experiences successive polarization rotations while propagating in the fiber. Each mode is depolarized differently with a random output polarization state, whose overlap with the desired output polarization is a random number between 0 and 1. When light is launched into all spatial modes of the fiber, the probability for all of them to have a substantial overlap with the desired polarization state at the output decreases exponentially with N. Therefore, simultaneous control of the polarization states of all modes cannot be achieved by using spatial degrees of freedom alone.

To have the transmitted light in a desired polarization state, one should inject light only to the mode whose output polarization is the closest to the desired one. This reasoning can be shown mathematically. Since t_{HH} is now a diagonal matrix, the eigenvalues of $t_{\mathrm{HH}}^{\dagger}t_{\mathrm{HH}}$ are simply N independent random numbers with the n-th eigenvalue being the overlap between the output of the n-th mode and the desired polarization state. The eigenvector with the largest eigenvalue corresponds to sending light only into the mode whose output polarization has the largest overlap with the desired polarization. The joint probability density of the N eigenvalues is simply $p(\tau_1,\ldots,\tau_N)=\prod_{n=1}^{N}p(\tau_n)=1$, and the probability density of the maximal eigenvalue [as given by Eq. (S2)] is

$$p\prime(\tau_{\max})=N\int_0^{\tau_{\max}}p(\tau_1)d\tau_1\cdots\int_0^{\tau_{\max}}p(\tau_{N-1})d\tau_{N-1}. \qquad (5)$$

It follows that

$$p\prime(\tau_{\max})=N(\tau_{\max})^{N-1},\quad \tau_{\max}\in(0,1). \qquad (6)$$

From it we have that $\langle\tau_{\max}\rangle=1-1/(N+1)$ and PER $=\langle\tau_{\max}\rangle/(1-\langle\tau_{\max}\rangle)=N$.

The above results are obtained without loss in the fiber. When the fiber has significant loss, $\langle\tau_{\max}\rangle$ is reduced. So is the PER, as will be shown in section VIII.

VI. FIBER REFRACTIVE INDEX PROFILE

The refractive index profile of the MMF whose data are presented in the main text is designed to reduce mode-

dependent loss. The core diameter is 50 μm and the measured refractive index profile is plotted in Fig. S3. A sharp drop of the refractive index at the interface between the core and the cladding enhances optical confinement and reduces the probability of light escaping from the core.

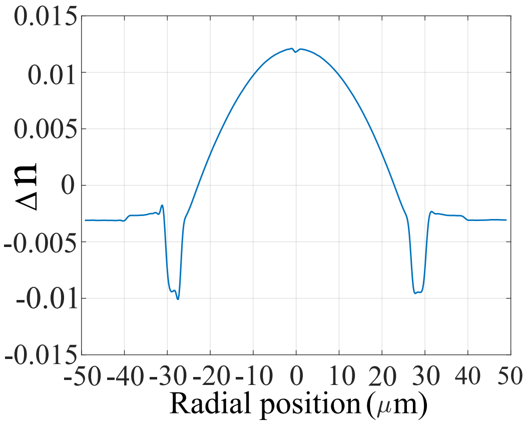

FIG. S3: Measured refractive index profile of the multimode fiber. The difference between the refractive index in the core and that in the cladding, Δn, has a parabolic profile within the core (from -25 μm to 25 μm), and a sharp drop at the interface between the core and the cladding to reduce light leakage.

VII. MODE COUPLING

Experimentally we use clamps to apply stress to the fiber. To confirm that mode coupling occurs in the fiber, we measure the output intensity patterns with and without clamps, when a plane wave is launched into the fiber at normal incidence. As shown in Fig. S4, the output intensity pattern is speckled, indicating the output field consists of multiple LP modes. When the fiber is pressed by the clamps, the number of speckle grains increases and the speckle grain size decreases. Therefore, more spatial modes are excited in the fiber due to the enhanced mode mixing introduced by the clamps.

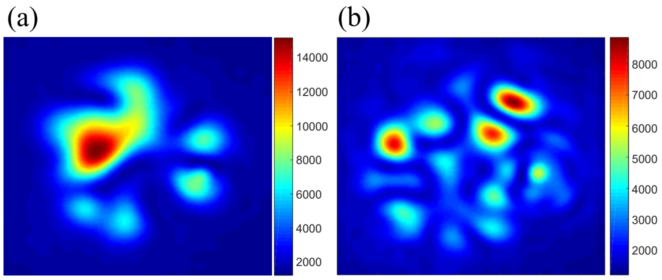

FIG. S4: Output intensity patterns for an MMF with (a) or without (b) clamps applying stress to it. A plane wave is launched into the fiber at normal incidence. The clamps enhance mode mixing in the fiber.

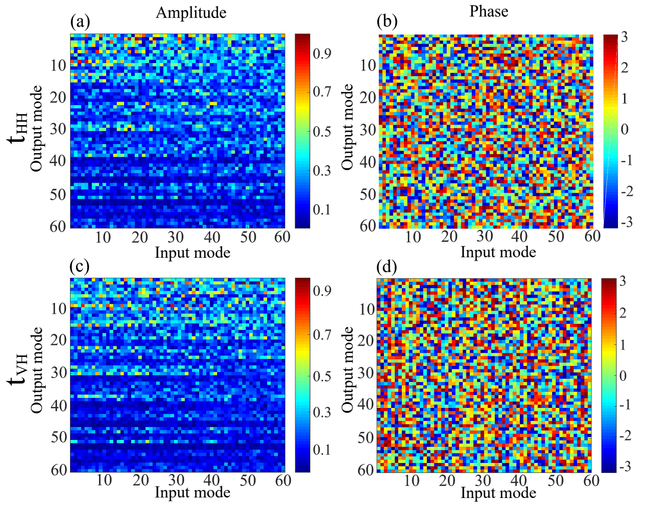

FIG. S5: Field transmission matrices of the MMF at $\lambda = 1550$ nm. Amplitude (a,c) and phase (b,d) of the measured $t_{\rm HH}$ and $t_{\rm VH}$. $t_{\rm HH}$ in (a,b) has both input and output horizontally polarized. $t_{\rm VH}$ in (c,d) has input horizontally polarized and output vertically polarized.

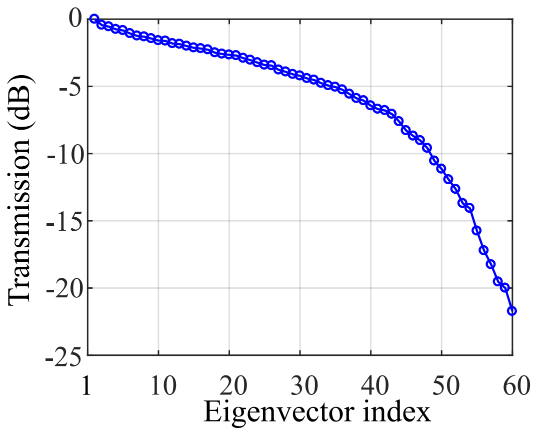

FIG. S6: Eigenvalues of the measured matrix $t_{\rm H}^{\dagger} t_{\rm H}$ of the MMF. The eigenvalues are normalized by their maximum. The decay of $\tau_{\rm HH}$ indicates mode-dependent loss in the fiber.

As described in the main text, the transmission matrix is measured with input in momentum (wavevector) basis and output in real space, and we perform a basis transform to represent the matrix in the fiber LP mode basis. Figure S5 presents experimentally measured field transmission matrices, $t_{\rm HH}$ and $t_{\rm VH}$, of the MMF for two output polarizations H and V, with the input polarization set to H. The eigenvalues of $t_{\rm H}^{\dagger} t_{\rm H}$, in which $t_{\rm H} = \binom{t_{\rm HH}}{t_{\rm VH}}$, reveals the mode-dependent loss in the fiber. As seen in Fig. S6, the sharp drop of the transmission curve after mode 50 indicates the cut-off of guided modes in the fiber.

The measured transmission matrix, represented in LP

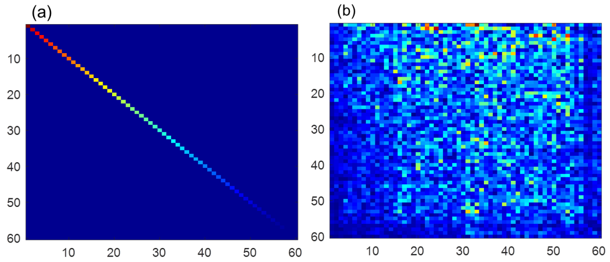

FIG. S7: Experimentally measured $t_{\rm HH}$ and $t_{\rm VH}$ cannot be made diagonal by a common basis transform. Singular value decomposition of $t_{\rm HH} = U\Sigma V^{\dagger}$. (a) The diagonal matrix Σ. (b) The transformed matrix $U^{\dagger} t_{\rm VH} V$ is non-diagonal. A common basis transformation cannot diagonalize $t_{\rm HH}$ and $t_{\rm VH}$ simultaneously.

mode basis, shows that with input light in a single LP mode, the fiber output spreads over all LP modes. To quantify the degree of mode coupling, we calculate the inverse participation ratio (IPR) of the LP modes constituting the fiber output field when the input light is in a single LP mode. The IPR is defined as IPR $\equiv (\sum_{i=1}^{60} P_i)^2 / \sum_{i=1}^{60} {P_i}^2$, where P_i is the intensity of the i-th LP mode at the fiber output. If the output field consists of only one LP mode, the IPR is equal to 1. If the output is a random superposition of all LP modes (with statistically equal weight), the IPR is 30. In our experiment, the IPR of the output field for a single LP mode input is about 22, indicating the fiber output contains far more than one LP mode due to strong mode mixing in the fiber. The IPR is lower than 30 because the higher order modes experience more loss and have lower output intensities than the lower-order ones.

A systematic error in transforming the transmission matrix from the measurement basis to the LP mode basis might make the measured $t_{\rm HH}$ and $t_{\rm VH}$ non-diagonal matrices. If there were the case, a common basis transformation could diagonalize both $t_{\rm HH}$ and $t_{\rm VH}$. To test this possibility, we conduct the singular value decomposition of the measured $t_{\rm HH}$. The input and output singular vectors of $t_{\rm HH}$ diagonalize $t_{\rm HH}$, but they cannot diagonalize the measured $t_{\rm VH}$, as shown in Fig. S7. This result confirms the measured transmission matrix corresponding to that of an MMF with strong mode and polarization coupling.

VIII. MODE-DEPENDENT LOSS

In the MMF, the lower order modes experience less attenuation than the higher order modes. To simulate the mode-dependent loss, we assume the decay length ξ for each LP mode is proportional to its propagation constant β, $\xi = \gamma\,\beta$, where γ is a coefficient. By varying the value of γ, we can tune the amount of loss. We solve for the eigenvectors of $t_{\rm HH}^{\dagger} t_{\rm HH}$ with the maximum eigenvalues and compute the PER. Fig. S8(a) plots the PER as a function of γ. The stronger the MDL, the lower the PER. Hence, the MDL reduces the polarization control.

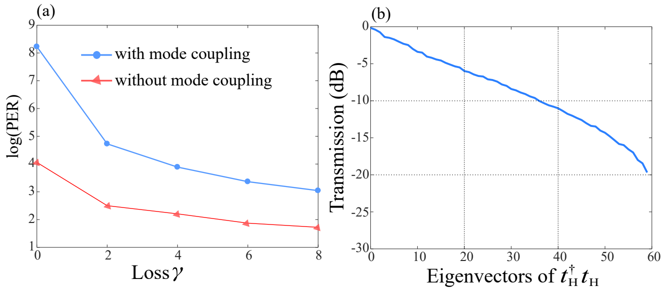

FIG. S8: Polarization control in the presence of MDL. (a) PER for the output field of the eigenvector with the maximum eigenvalue of $t_{\rm HH}^{\dagger} t_{\rm HH}$ as a function of the MDL, characterized by γ, for the MMF with (blue dot) and without (red triangle) mode coupling. (b) Eigenvalues of the numerical $t_{\rm H}^{\dagger} t_{\rm H}$ for γ = 6, normalized by the maximum value. The range of decay is comparable to that of the fiber used in our experiment in Fig. S6.

Mode-dependent loss also reduces the polarization control when there is no mode mixing in the fiber. We repeat the calculation for the MMF without mode mixing, and obtain much lower PER for the same amount of MDL. Fig. S8(a) shows no matter how strong the MDL is, the PER without mode coupling is always lower than that with mode coupling. Therefore, mode coupling enhances the polarization control even when the fiber has MDL.

We adjust the amount of MDL in the numerical simulation to match that of the fiber in the experiment. Figure S8(b) plots the eigenvalues of numerically calculated t_H, which decay over a range comparable to those of the experimentally measured t_H in Fig. S6. With this amount of MDL in the numerical model, the eigenvector with the maximum eigenvalue of $t_{\rm HH}^{\dagger} t_{\rm HH}$ has a PER of 29, which is close to the experimental PER of 24. Without mode mixing, the PER is found numerically to be 6.5, much lower than the PER with mode mixing.

IX. WAVELENGTH DEPENDENCE

In the main text we demonstrate polarization control in an MMF for monochromatic light, which is often used for optical excitation in polarization-resolved fluorescence microscopy. While our scheme of polarization control works for any wavelength, we must adjust the input wavefront with wavelength in order to realize a specific output polarization state. This is because the transmission matrix $t_{\rm HH}$ is wavelength dependent, and its spectral correlation width is determined by the properties of the fiber, such as the length, the differential group delay and the degree of mode mix-

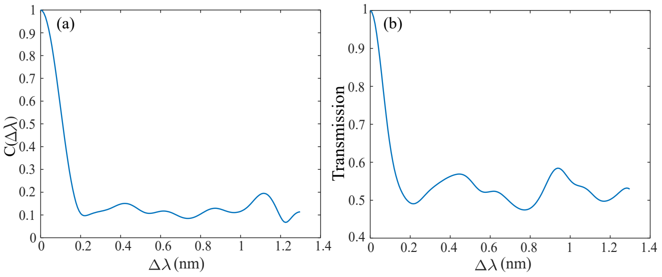

FIG. S9: (a) Spectral correlation function $C(\Delta\lambda)$ of an MMF with strong mode coupling and negligible loss. (b) Wavelength dependent transmission in horizontal polarization for the eigenvector with largest eigenvalue of $t_{\mathrm{HH}}(\lambda_0)$.

ing. The spectral correlation function is defined as $C(\Delta\lambda) = \langle I(\lambda_0)I(\lambda_0+\Delta\lambda)\rangle/\langle I(\lambda_0)\rangle\langle I(\lambda_0+\Delta\lambda)\rangle - 1$, where $I(\lambda)$ is the output intensity pattern at wavelength λ for a fixed input wavefront. We compute $C(\Delta\lambda)$ using $t_{\mathrm{HH}}(\lambda)$ for 1550 nm $\leqslant \lambda \leqslant$ 1551.3 nm in the numerical simulation of an MMF with strong mode mixing but negligible loss. The spectral width of $C(\Delta\lambda)$ is 0.2 nm in Fig. S9(a). We further calculate the eigenvector with the largest eigenvalue of $t^{\dagger}_{\mathrm{HH}}t_{\mathrm{HH}}$ at $\lambda_0 = 1550$ nm. The transmission of horizontal polarization for this eigenvector is unity at λ_0, as expected. As shown in Fig. S9(b), when the wavelength λ is detuned from λ_0 by $\Delta\lambda$, the transmission in horizontal polarization decreases and the transmission in vertical polarization increases, eventually both approach 0.5 for $\Delta\lambda \geqslant 0.2$ nm. Thus the bandwidth of the polarization-preserving channel is equal to the spectral correlation width of the fiber. The same bandwidth is found for the polarization-changing channels. For the applications in nonlinear optics, broad-band pulses are usually used, and a large bandwidth of the polarization-shaping channels is required. This can be achieved by using MMFs with small differential group delay, which gives a large spectral correlation width.

X. MULTI-CHANNEL POLARIZATION CONTROL

As stated in the main text, wavefront shaping can convert arbitrary polarization states of the input light to any arbitrary polarizations of individual modes at the output of a multimode fiber with strong mode and polarization mixing. Here we illustrate how to realize this with an example. First we construct the transmission matrix that relate different input and output polarization states. We calculate t_{HH}, t_{VH}, t_{HV}, t_{VV} of a fiber with 60 spatial modes using the concatenated fiber model. From them, we construct, e.g., the transmission matrix for linear vertical (V) polarization input and left-circular (L) polarization output $t_{\mathrm{LV}} = (1/\sqrt{2})(t_{\mathrm{HV}} + it_{\mathrm{VV}})$, or the transmission matrix for right-circular (R) polarization input

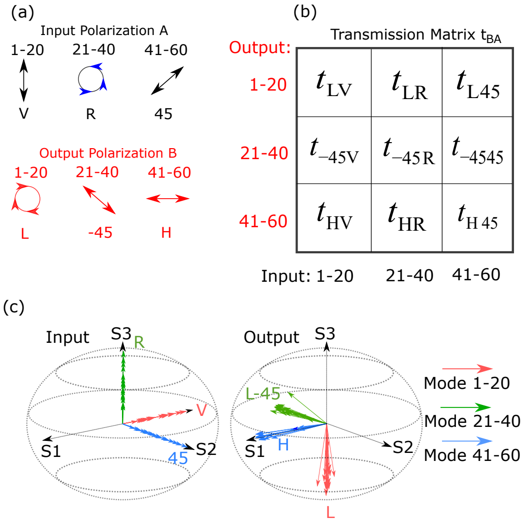

FIG. S10: Multi-channel polarization control. (a) Input polarization state A and output polarization state B. Modes 1-20 have the linear vertical (V) polarization at the input and the left-circular (L) polarization at the output. Modes 21-40 have the right-circular (R) polarization at the input and the linear $-45°$ polarization at the output. Modes 41-60 have the linear 45° polarization at the input and the linear horizontal (H) polarization at the output. (b) Corresponding transmission matrix t_{BA} is constructed. (c) The Poincaré sphere representation of the input and output polarization states for all modes of the eigenvector of $t^{\dagger}_{\mathrm{BA}}t_{\mathrm{BA}}$ with the largest eigenvalue.

and L polarization output $t_{\mathrm{LR}} = (1/\sqrt{2})(t_{\mathrm{LH}} - it_{\mathrm{LV}})$, as described in the main text. Next we consider individual input and output modes have different polarizations, e.g., let us bin the LP modes into three groups, i.e. 1-20, 21-40 and 41-60. As illustrated in Fig. S10(a), the input polarization state A has V polarization for modes 1-20, R polarization for modes 21-40 and linear 45° (45) polarization for modes 41-60. The output polarization state B is designed to be L polarization for modes 1-20, linear -45° (-45) polarization for modes 21-40 and horizontal (H) polarization for modes 41-60. To achieve the conversion from A to B, we construct the corresponding transmission matrix t_{BA} shown in Fig. S10(b). For example, the elements for 1-20 rows and 1-20 columns of t_{BA} are a copy of the elements in 1-20 rows and 1-20 columns of t_{LV}, the elements of 1-20 rows and 21-40 columns of t_{BA} are copied from the corresponding rows and columns of t_{LR}, etc. We then compute the eigenvalues and eigenvectors of $t^{\dagger}_{\mathrm{BA}}t_{\mathrm{AB}}$. When the fiber has no loss, the maximum eigenvalue is equal to 1. Figure S10(c) presents the Poincaré sphere representation of the input (left) and output (right) polarization states of the corresponding eigenvector. The arrows of three colors (red, green and blue) denote the polarization states for three groups of LP modes (1-20, 21-40 and 41-60). These results confirm that the eigen-

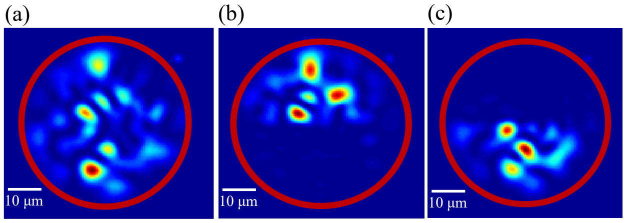

FIG. S11: Output intensity pattern (a), its left-hand (b) and right-hand (c) circularly polarized components reveal the transmitted field in the top half of the fiber facet is the left circularly polarization, and the bottom half right circularly polarization.

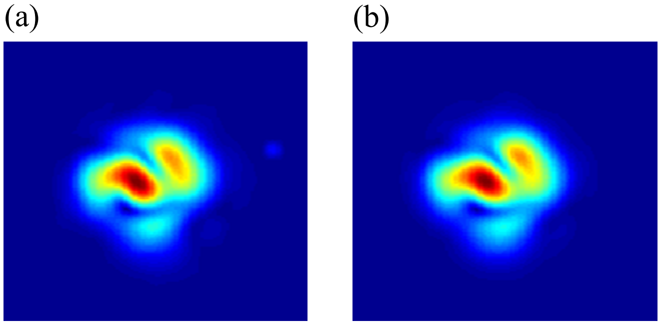

FIG. S12: Output intensity patterns of an eigenstate of $t_{\rm HH}$, which retain the input polarization state (linear horizontal). (a) The pattern predicted by the measured transmission matrix. (b) The pattern measured experimentally after shaping the input wavefront. Their agreement confirms the experimental stability.

vector of $t_{\rm BA}^{\dagger} t_{\rm BA}$ with the unity eigenvalue has the input polarization state A and the output polarization state B, thus realizing the polarization conversion from A to B.

Figure S11 shows an numerically generated polarization state using the experimentally measured transmission matrices $t_{\rm HH}$ and $t_{\rm VH}$. All spatial channels in the top half of the fiber facet are left-hand circularly polarized (L) and the bottom half right-hand circularly polarized (R).

XI. EXPERIMENTAL STABILITY

In the experiment, the 2m long bare fiber is coiled to 5 loops (not on a spool) and pressed by 12 clamps. The clamps not only introduce mode coupling in the fiber but also stabilize the fiber. The effect of temporal drift is negligible during the period of experiment. As a test, Fig. S12(a) shows the MMF output intensity pattern for an eigenstate retaining the input polarization, predicted by the measured transmission matrix. By modulating the amplitude and phase of input light and launching it into the fiber after the transmission matrix measurement, we record the output intensity pattern, as shown in Fig. S12(b). It agrees well with the predicted one, confirming the stability of the fiber during the period of the experiment.

[1] K. P. Ho and J. M. Kahn, Journal of lightwave technology **29**, 3119 (2011).
[2] C. W. Beenakker, Reviews of modern physics **69**, 731 (1997).
[3] H. U. Baranger and P. A. Mello, Phys. Rev. Lett. **73**, 142 (1994).
[4] R. A. Jalabert, J.-L. Pichard, and C. W. J. Beenakker, Europhys. Lett. **27**, 255 (1994).
